# Preferences for digital health services among patients with chronic kidney disease: a discrete choice experiment

**DOI:** 10.3389/fpubh.2026.1905738

**Published:** 2026-10-09

**Authors:** Lifang Wei, Yueyang Huang, Difei Duan, Yi Wen, Lu Yu, Xue Zhang, Jun Liu, Xindi Li, Dengyan Ma

**Affiliations:** 1 Department of Nephrology, West China Hospital of Sichuan University, Chengdu, Sichuan, China; 2 West China School of Nursing, Sichuan University, Chengdu, Sichuan, China; 3 Department of Nephrology, Meishan People's Hospital, Meishan, Sichuan, China; 4 Department of Nephrology, Nanchong Gaoping District People's Hospital, Nanchong, Sichuan, China; 5 Department of Nephrology, University-Town Hospital of Chongqing Medical University, Chongqing, China; 6 Department of Nephrology, Xinjiang Medical University Affiliated Traditional Chinese Medicine Hospital, Xinjiang, China

**Keywords:** China, chronic kidney disease, digital health services, discrete choice experiment, preference

## Abstract

**Background:**

Digital health technologies offer novel approaches for chronic kidney disease management. However, quantitative evidence on patient preferences for digital health services remains limited. There is a critical need for more evidence on the sustainability and optimization of chronic kidney disease management in China, especially from the demand side. Policymakers require detailed data on the needs of chronic kidney disease patients regarding digital health services.

**Methods:**

A discrete choice experiment was conducted to measure the preferences of people with chronic kidney disease. Participants were recruited face-to-face in Sichuan Province, China, and completed an anonymous electronic questionnaire on-site. The mixed logit model evaluated participants’ preferences for six attributes, estimating willingness to pay and relative importance, and performing subgroup analysis based on the initial model results.

**Results:**

A total of 322 patients completed the questionnaire (mean age 49.8 years; 55.3% female). Out-of-pocket cost was the most influential attribute (relative importance 50.8%), followed by healthcare provider type (17.2%) and consultation frequency (10.0%). Patients preferred the combination of an attending nephrologist and a clinical nurse specialist (coefficient = 0.716; 95% CI, 0.510–0.921) and a higher consultation frequency (coefficient for five consultations per month = 0.414; 95% CI, 0.208–0.620). Willingness to pay for the combined provider team was 52.99 yuan. Exploratory subgroup analyses suggested possible differences in preferences by sex, age, disease duration, and comorbidity type, but most formal between-group comparisons were not statistically significant and were unadjusted for multiple testing.

**Conclusion:**

Out-of-pocket cost is the primary determinant of digital health service selection among patients with chronic kidney disease. The type of healthcare provider is critical for service appeal. Exploratory subgroup analyses suggested possible preference differences across patient groups, indicating that stratified or preference-sensitive service designs may merit further investigation. Future research should examine whether aligning digital health services with patient preferences is associated with improved engagement or outcomes in real-world settings.

## 1 Introduction

Chronic kidney disease (CKD) is a progressive and irreversible decline in kidney function that may eventually lead to end-stage renal disease and significantly increases the risk of cardiovascular events and death (1–3). According to the Global Burden of Disease Study, the global prevalence of CKD continues to rise, posing a major public health challenge (4). In China, the estimated prevalence of CKD among adults is 8.2%, affecting over 82 million people and imposing substantial economic and social burdens (5). Patients with CKD require long-term monitoring of kidney function, medication adjustments, and management of complications (6–8). Traditional care, which revolves around hospitals and relies on regular outpatient follow-up, often suffers from poor continuity, delayed responses, and low patient engagement (9).

Digital health technologies offer new possibilities for CKD management by shifting care from a passive, episodic model to an active, continuous intervention approach (10, 11). Common forms include self-management applications, remote monitoring, tele-nutrition counseling, and wearable devices (12–14). However, most of these services are designed from a provider perspective, focusing on technical feasibility and effectiveness, with limited exploration from the patient’s viewpoint (15). As end users, patients’ preferences directly determine the attractiveness and accessibility of digital health services. Therefore, accurately measuring patient preferences is crucial for developing effective management strategies.

Previous qualitative studies indicate that CKD patients highly value disease-related knowledge acquisition, dietary and medication guidance, and privacy protection features (16–18). Educational content receives the highest engagement, while passive recording functions (e.g., symptom tracking) are used less frequently (19). Patients also tend to prefer a hybrid model combining virtual and face-to-face interactions (20). Nevertheless, when facing trade-offs among different service attributes—such as follow-up frequency, communication mode, out-of-pocket costs, and provider type—how patients weigh and make choices remains unclear due to a lack of quantitative evidence.

In healthcare services, preference refers to the degree of individuals’ cognitive inclination toward different health conditions and service offerings, encompassing values related to physiological, psychological, emotional, and social support. Common methods for measuring preferences include the contingent valuation method, conjoint analysis, and discrete choice experiments (DCE). The contingent valuation method can only assess preference through trade-offs between two attributes, which limits its scope (21). Conjoint analysis requires strict mathematical derivation and proof; moreover, it has limitations in explaining preference or choice behavior because it does not capture the actual utility that healthcare services provide to individuals (22). In contrast, discrete choice experiments allow for a comprehensive evaluation of multiple attributes, face relatively weaker hypothetical bias, and closely mimic real-world choices. The preferences derived from discrete choice experiments can effectively predict actual behavior and elucidate individuals’ trade-offs across multiple attributes, making them widely applicable in healthcare service research (23).

Given these advantages, the DCE serves as a reliable preference-elicitation method. It evaluates multiple attributes in a simulated real-choice context and demonstrates good predictive validity for actual behavior (23, 24). DCE draws on demand theory and random utility theory: people’s demand depends on the combination of service attributes, and they select the option that maximizes their utility. In a DCE, respondents choose among hypothetical scenarios with varying attribute levels, allowing researchers to quantify their preferences and trade-offs. This approach helps identify service designs that best align with patient expectations from the demand side. While nephrology research has used DCE to explore preferences for kidney graft acceptance, care settings, and dialysis modalities, quantitative studies focusing on digital health services for chronic kidney disease (CKD) remain rare (25–28). Therefore, this study employs a DCE to measure CKD patients’ preferences for digital health services, aiming to inform the optimization of digital health management programs for CKD. Additionally, these insights will offer valuable perspectives for regions facing similar healthcare challenges.

## 2 Materials and methods

This study applied the DCE according to the ISPOR Conjoint Analysis Good Research Practices and the WHO guidelines for using discrete choice models (22, 29). The DCE design mainly includes four stages: (1) design of attributes and levels; (2) experimental design; (3) questionnaire design and data collection; (4) analysis and interpretation of the data.

### 2.1 Design of attributes and levels

The attributes and levels included in the DCE were identified through a targeted literature review and expert consultation. The literature search was conducted in January 2025, and the expert consultation was carried out between February and May 2025. The targeted literature review was performed to identify attributes relevant to patient preferences for digital health services in CKD management; it was intended to generate a candidate pool of attributes rather than to serve as a comprehensive systematic review.

We searched six major English-language databases—PubMed/MEDLINE, Embase, Cochrane Library, Web of Science, CINAHL, and Scopus—from inception to January 2025. The search strategy combined terms related to three domains: (i) digital health technologies (e.g., “digital health,” “digital health technology,” “mHealth,” “remote monitoring”); (ii) chronic kidney disease (e.g., “chronic kidney disease,” “renal insufficiency,” “kidney failure,” “dialysis”); and (iii) patient preferences (e.g., “patient preference,” “discrete choice experiment,” “conjoint analysis”). Studies were considered relevant if they were peer-reviewed English-language studies that elicited patient preferences for digital health services in CKD. Editorials, conference abstracts, and studies not reporting original preference data were excluded. Titles and abstracts were screened for relevance, and full texts of potentially relevant studies were reviewed.

Five highly relevant articles were included in the analysis, from which eight potential attributes were identified (13, 18, 30–32): (1) monitoring, (2) healthcare provider, (3) online community interaction, (4) AI-based health services, (5) number of consultations, (6) monthly out-of-pocket costs, (7) response time, and (8) supplementary services.

In accordance with DCE design principles, which recommend no more than six attributes with two to four levels per attribute to maintain statistical efficiency and respondent feasibility (33), a panel discussion was convened following the targeted literature review. The panel comprised 10 experts, including three health economics specialists, three health policy experts, two nursing specialists with over a decade of clinical experience in CKD management, one digital health technology expert, and one patient organization representative for individuals with CKD. Preliminary definitions and proposed levels for each attribute were presented, and panel members were invited to evaluate the importance and feasibility of each attribute and level. Experts were also encouraged to suggest additional relevant attributes.

Following expert consultation, two attributes—response time and supplementary services—were excluded due to perceived low relevance or redundancy with other attributes. No new attributes were proposed. Six experts noted consensus that supplementary services and AI-based health services exhibited content overlap; given that AI-based health services were considered more aligned with current patient needs, supplementary services were removed from consideration. Five experts suggested that response time, defined as the interval until healthcare providers responded to patient inquiries, was of secondary importance relative to consultation frequency and provider type and was therefore removed after comprehensive consideration.

All attribute levels were reviewed by the expert panel and judged to be realistic for Chinese patients with CKD receiving digital health services. To enhance comparability across respondents with differing clinical and economic conditions, monthly out-of-pocket costs and the number of online consultations were defined with explicit reference points and presented as discrete levels. Monthly out-of-pocket costs were expressed as 50 yuan, 100 yuan, and 200 yuan, reflecting the typical patient-borne expenses for digital health services in CKD, including remote monitoring subscriptions, online consultations, and related digital care management in China; these levels were derived from published cost data and confirmed by the panel as representing realistic and salient amounts under current digital health practice (34). The number of online consultations was defined as the number of scheduled online follow-up contacts per month and was set at 1, 3, and 5 times. These frequencies were based on current digital health care patterns and panel consensus (35).

For the AI-powered health services attribute, levels were operationalized as binary categories but were presented to respondents using concrete functional descriptions rather than evaluative labels alone. The “good” level was described as: “People could access an AI-powered conversation service to consult about their condition and health management,” whereas the “poor” level was described as a service without this AI conversation feature. The remaining attributes—monitoring, healthcare provider, and online community interaction—were described using categorical levels. Monitoring and online community interaction each included two levels, while healthcare provider type included three levels (see Supplementary Table S1).

### 2.2 Experimental design

Based on the six attributes and their corresponding levels, a full factorial design generated 216 (2^3^ × 3^3^ = 216) hypothetical scenarios and 23,220 [(216 × 215)/2] paired-choice tasks, which was deemed impractical for respondents (36). To enhance practical applicability and reduce respondents’ cognitive burden, a fractional factorial D-efficient design with zero priors was generated using Stata version 16.0, producing 16 choice sets with a D-error of 0.95 (37, 38). The design aimed to satisfy orthogonality, level balance, and minimal overlap and focused on estimating main effects under the assumption of negligible interactions between attributes (39). To further mitigate respondent cognitive load, the 16 choice sets were divided into two versions of the DCE questionnaire (40). Participants were randomly assigned to complete one version containing eight DCE choice sets. To assess response consistency and rationality, two test items were incorporated (41). Test item (1) comprised a combination of an optimal choice and other alternatives, while test item (2) was identical to choice task 2. Consequently, each participant responded to a total of 10 choice tasks. Respondents who failed to select the optimal option in test item (1) or provided inconsistent responses between test item (2) and task 2 were considered for exclusion from the final analysis. An example DCE choice set is presented in Table 1.

**Table 1:** Example of a DCE set.

Attribute	Option A	Option B
Monthly out-of-pocket expenses	50 yuan 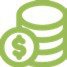	200 yuan 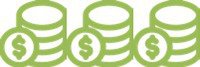
Healthcare provider	Attending nephrologist 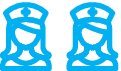	Clinical nurse specialist 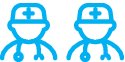
Number of consultations	5 times 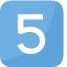	3 times 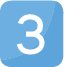
AI-powered health services	Good 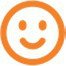 : People could access an AI-powered conversation service to consult about their condition and health management	Poor 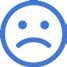 : A service without an AI conversation feature
Monitoring	Manual entry 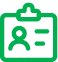	Remote monitoring 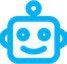
Online community interaction	Yes 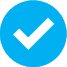	No 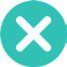
Your choice:	Option A □	Option B □

### 2.3 Questionnaire design and data collection

The final questionnaire comprised two sections: demographic characteristics, including age, sex, marital status, educational attainment, monthly income level, disease duration, treatment history, and lifestyle factors; and a series of DCE choice sets. Specifically, participant eligibility criteria included: (1) having a confirmed clinical diagnosis of CKD according to the KDIGO clinical practice guideline criteria (42), based on abnormalities of kidney structure or function persisting for >3 months, documented in medical records and supported by laboratory and clinical data; CKD stage was classified as G1–G5, with G1–G2 requiring albuminuria (ACR ≥ 30 mg/g) or another kidney damage marker, and G5 restricted to non-dialysis patients who had not initiated kidney replacement therapy (dialysis or kidney transplantation) (43); (2) age ≥18 years; (3) adequate reading comprehension to understand questionnaire content and complete choice tasks independently; and (4) voluntary participation. Exclusion criteria were: (1) current receipt of kidney replacement therapy (dialysis or kidney transplantation) or a functioning kidney transplant; (2) severe comorbidities that would prevent completion of the questionnaire or substantially affect treatment preferences (e.g., end-stage malignancy, severe heart failure); (3) acute kidney injury or potentially reversible kidney function impairment; and (4) cognitive or perceptual dysfunction, or inability or unwillingness to provide informed consent.

A pilot test was conducted with 20 patients with CKD prior to the main survey. Completion times were recorded, and brief debriefing interviews were conducted immediately after task completion. Most participants accurately understood the attribute levels, but some suggested that the description of the AI-powered health services attribute could be further clarified. Based on this feedback, supplementary explanations were added to the relevant attribute descriptions before the main survey. Participants also confirmed that the number of choice tasks was reasonable and did not impose an excessive time burden. Because the pilot test was intended to assess comprehensibility and feasibility rather than to obtain parameter estimates for design revision, the zero-prior design was retained unchanged. Data from the pilot test were excluded from the final analysis.

We used the Rules of Thumb to calculate the minimum sample size required for the DCE main effects model (36, 44). The sample size depends on three factors: the levels of the DCE attributes, the number of choice sets in the DCE, and the number of options in each choice set, as follows:

$$N>\frac{500\times c}{\left(t\times a\right)}$$

Where 500 is a constant, c is the maximum number of levels in any attribute, t represents the number of DCE options in each questionnaire, and a refers to the number of options in each option. After calculation, the sample size of this study was 94. However, we increased the sample size to improve the precision of estimates for both the overall sample and subgroups.

Participants were recruited face-to-face by trained research staff during routine follow-up visits at a hospital-based, nurse-led outpatient clinic dedicated to pre-dialysis CKD follow-up (the CKD Whole-Course Management Center). After providing informed consent, participants completed an anonymous electronic questionnaire on-site using the Wenjuanxing platform (www.wjx.cn). Participants were randomly assigned to one of the two DCE questionnaire versions using the page-splitting function of the Wenjuanxing platform. Participants were instructed to carefully weigh each set of choices and select their preferred option by checking the corresponding box for either Option A or Option B. Each questionnaire could be submitted only once per device to prevent duplicate entries. The survey included two test choice sets at the beginning to assess internal consistency; respondents failing these consistency checks were excluded from analysis. Questionnaires with straight-lining responses, defined as selecting the same alternative position (e.g., always the first option) across all choice sets, were also excluded (45). Informed consent was obtained from all participants prior to data collection.

### 2.4 Analyze and interpret the data

All statistical analyses were performed using Stata version 16.0. Sociodemographic characteristics of patients with CKD were summarized using descriptive statistics. DCE data were analyzed using mixed logit models. The mixed logit model accommodated preference heterogeneity across the sample by specifying cost as a fixed parameter, while all remaining attributes were specified as random parameters.

All attribute levels, including monthly out-of-pocket expenses, were dummy coded to allow for potential non-linear effects on participants’ choices. The mixed logit model was estimated using simulated maximum likelihood with 500 Halton draws. The random-parameter specification was pre-specified: the coefficient for monthly out-of-pocket expenses was specified as a fixed parameter to facilitate estimation of willingness-to-pay, whereas the coefficients for all other attributes were specified as random parameters and assumed to follow independent normal distributions to capture preference heterogeneity across participants (46). The panel structure was accounted for in the estimation, as the mixlogit command groups choice occasions by respondent and estimates the likelihood at the individual level. The utility U for the respondent i choosing an alternative j in the choice task t was specified as follows:

$$U_{\mathrm{ijt}\;\;}=V_{\mathrm{ijt}}(X_{\mathrm{ijt}},\beta_{i})+\varepsilon_{\mathrm{ijt}}$$

where $V_{\mathrm{ijt}}$ represents the fixed component of utility, $X_{\mathrm{ijt}}$ is a vector of attribute levels for the alternative, $\beta_{i}$ denotes the vector of coefficients, and $\varepsilon_{\mathrm{ijt}}$ represents the error term. Positive (negative) β coefficients indicated whether an attribute level combination was preferred (not preferred) relative to the reference level. Larger absolute β values reflected stronger preferences for that level relative to the reference category. Statistical significance was determined when the 95% confidence intervals for both the mean relative utility ( β ) and standard deviation (SD) excluded zero. The mixed logit model converged normally. A conditional logit model was also estimated as a robustness check, and Akaike Information Criterion (AIC) and Bayesian Information Criterion (BIC) were used to compare model fit between the mixed logit and conditional logit models (46). No formal comparison of alternative random-parameter specifications within the mixed logit model was performed.

Relative importance scores quantified the weight of each attribute, calculated as the difference in utility between the lowest and highest levels of a given attribute divided by the sum of utility differences across all attributes (22).

To estimate willingness-to-pay, a single marginal utility of cost is required. Because monthly out-of-pocket expenses were dummy-coded in the primary mixed logit model, we estimated an additional constrained mixed logit model in which cost was treated as a continuous linear variable, with all other model specifications unchanged. The appropriateness of the linear cost assumption was assessed using a likelihood ratio test. Willingness-to-pay for each attribute was calculated as the negative ratio of the attribute coefficient to the cost coefficient (−β_attribute/β_cost) (28, 47). Standard errors and 95% confidence intervals were obtained using the delta method via the wtp command in Stata.

Using the estimated mixed logit model, we simulated individual-specific choice probabilities for alternative scenarios and averaged them across the sample. For each scenario, all other attributes were held constant at baseline levels while one attribute was changed at a time. Reported values represent absolute percentage-point changes in predicted choice probability, calculated as (P_alternative − P_baseline) × 100.

Subgroup analyses were conducted using mixed logit models stratified by demographic and clinical characteristics, including sex (male vs. female), age (< 45, 45–60, ≥60 years), marital status (married vs. unmarried), disease duration (< 6 vs. ≥6 years), and comorbidity categories. Comorbidity groups were mutually exclusive. Patients were classified according to physician-documented clinical diagnoses in the hospital information system (HIS). When multiple comorbidities were documented and no primary comorbidity was specified in the clinical record, a prespecified hierarchy (cardiovascular disease > diabetes > hypertension) was applied (42, 48). Willingness-to-pay estimates were also calculated for each subgroup. Seemingly unrelated regression estimation was used to compare regression coefficients across subgroups.

All statistical analyses were conducted using Stata version 16.0 (mixlogit and mixlpred commands). Statistical significance was defined as two-tailed *p* < 0.05.

## 3 Results

### 3.1 Participant characteristics

A total of 427 patients with CKD completed the electronic questionnaire. After excluding participants who failed the dominance test (*n* = 71) or provided straight-line responses (*n* = 34), 322 respondents (75.4%) were included in the final analysis. The two questionnaire versions were completed by 169 and 157 respondents, respectively, with no significant differences in demographic characteristics between versions (*p* > 0.05). Participant age ranged from 19 to 70 years (mean = 49.84, SD = 14.09). The majority of respondents were female (55.28%), married (69.25%), and had attained moderate educational levels (70.81%). Disease duration among respondents ranged from 1 to 28 years (mean = 6.07, SD = 5.82), and over half of the participants had comorbid hypertension (52.17%). Complete demographic characteristics of the study sample are presented in Table 2.

**Table 2:** Demographic characteristics of people with chronic kidney diseases (*N* = 322).

Characteristic		Value
Sex	Men	144 (44.72%)
	Women	178 (55.28%)
Age, years		49.84 ± 14.09
Marital status	Unmarried	99 (30.75%)
	Married	223 (69.25%)
Education level^a^	Low	9 (2.80%)
	Moderate	228 (70.81%)
	High	85 (26.40%)
Monthly income	≤3,000	114 (35.40%)
	3,001–6,000	106 (32.92%)
	6,001–9,000	65 (20.19%)
	≥9,000	37 (11.49%)
Disease duration, years		6.07 ± 5.82
Comorbidity categories	Type I	168 (52.17%)
	Type II	86 (26.71%)
	Type III	53 (16.46%)
	None of the above	15 (4.66%)
Travel time to the hospital, minutes	≤30	163 (50.62%)
	>30	159 (49.38%)

a The educational level was categorized into low (no education, primary education, or prevocational education), moderate (vocational education or selective secondary education), and high (university). Type I, CKD combined with hypertension; Type II, CKD combined with type 2 diabetes; Type III, CKD combined with cardiovascular diseases.

### 3.2 Patient preferences for digital health service attributes

A total of 2,576 choice observations from 322 respondents were included in the preference analysis.

In both the mixed logit and conditional logit models, all attribute levels were statistically significant relative to their reference levels, except for attending nephrologist alone (vs clinical nurse specialist), as shown in Supplementary Tables S2, S3. Because the mixed logit model had lower AIC (2756.340 vs. 2908.776) and BIC (2861.094 vs. 2967.700) values than the conditional logit model, it was selected as the primary specification and retained for subsequent analyses. As a sensitivity analysis, we re-estimated the mixed logit model using the full sample of 427 respondents, retaining both respondents who failed the dominance/consistency test and those with straight-line response patterns. The direction and statistical significance of most coefficients remained similar, and the overall preference hierarchy was unchanged (Supplementary Table S4). The main exception was remote monitoring, which became non-significant (*β* = 0.078, *p* = 0.212). The relative importance of healthcare provider type was also attenuated. Overall, the main findings were largely robust to the exclusion of these respondents.

For monthly out-of-pocket costs, both 100 yuan (*β* = −1.231, 95% *CI*, −1.408 to −1.055) and 200 yuan (*β* = −2.113, 95% *CI*, −2.340 to −1.885) were associated with significantly lower utility relative to 50 yuan, indicating a strong preference for lower costs. Among other attributes, remote monitoring (*β* = 0.326, 95% *CI*, 0.153 to 0.500), the combination of an attending nephrologist and a clinical nurse specialist (*β* = 0.716, 95% *CI*, 0.510 to 0.921), online community interaction (*β* = 0.190, 95% *CI*, 0.063 to 0.318), AI-powered health services (*β* = 0.401, 95% *CI*, 0.277 to 0.524), three consultations per month (*β* = 0.386, 95% CI, 0.217 to 0.555), and five consultations per month (*β* = 0.414, 95% *CI*, 0.208 to 0.620) were all significantly preferred over their respective reference levels. In contrast, receiving care from an attending nephrologist alone was not significantly different from receiving care from a clinical nurse specialist alone (*β* = 0.078, *p* = 0.400). The relative importance of the six attributes was ordered as follows: out-of-pocket costs (50.79%), healthcare provider type (17.21%), consultation frequency (9.95%), AI-based health services (9.64%), remote monitoring (7.84%), and online community interaction (4.57%).

Preference heterogeneity was assessed using the standard deviations of the random coefficients in the mixed logit model. Statistically significant heterogeneity was observed for the coefficients of remote monitoring (*SD* = 3.273, *p* < 0.001), attending nephrologist alone (*SD* = 1.719, *p* < 0.001), and five consultations per month (*SD* = 2.689, *p* < 0.001). In contrast, the standard deviations for the remaining random coefficients were not significantly different from zero (all *p* > 0.05), indicating limited preference variation for those attributes. Estimated coefficients and relative importance scores are presented in Figure 1.

**Figure 1:**
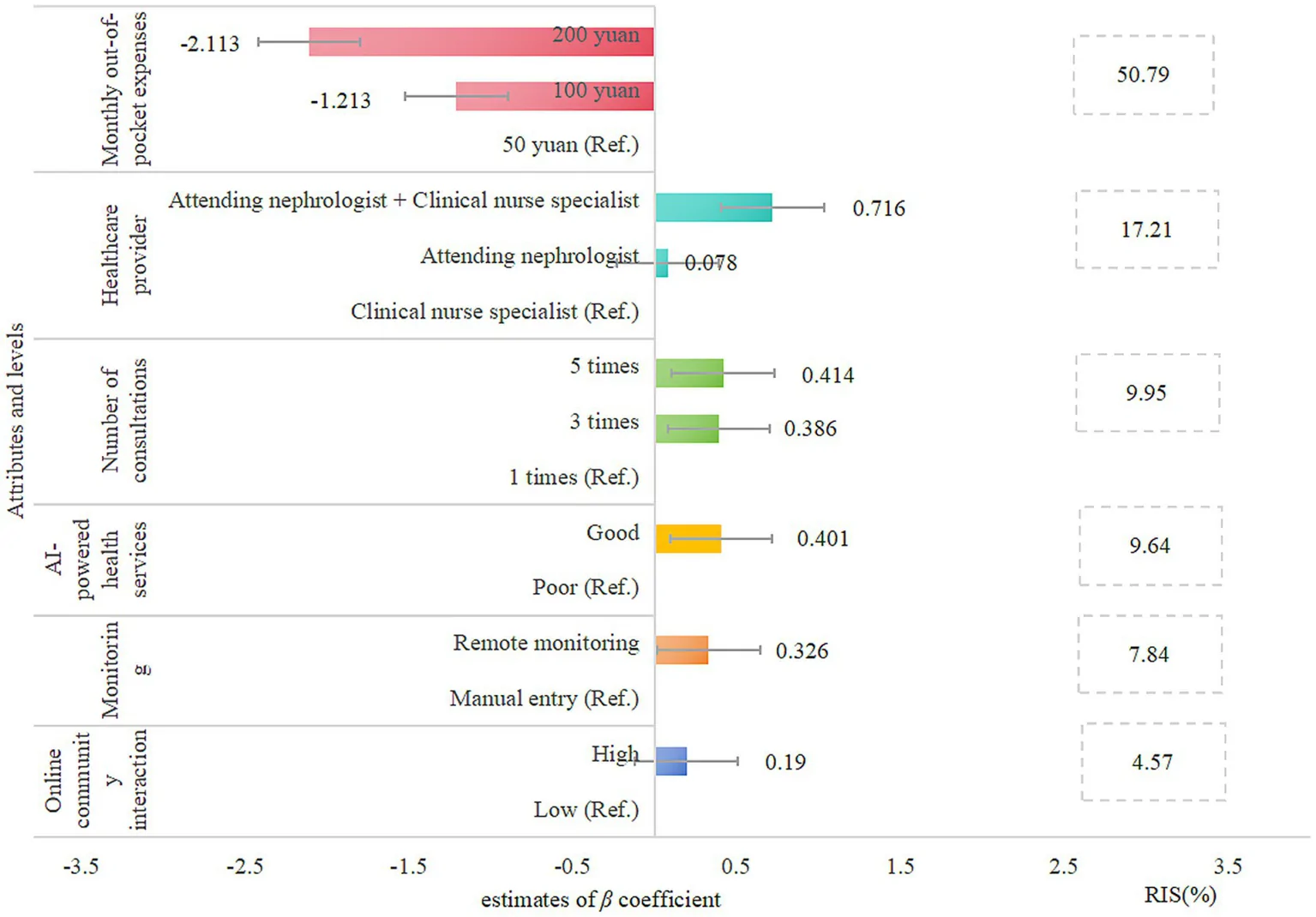
Estimation coefficients and relative importance scores of attributes in the mixed logit model.

### 3.3 Willingness-to-pay estimates

A separate constrained mixed logit model with cost as a continuous linear variable was estimated (see Supplementary Table S5). A likelihood ratio test comparing the categorical and continuous cost specifications did not indicate a significant worsening of fit when linearity was imposed (LR *χ^2^*(1) = 2.22, *p* = 0.136). Willingness-to-pay estimates are presented in Table 3. As model-based estimates derived from stated choices, these monetary values represent relative utility trade-offs rather than actual amounts that patients would necessarily pay in real-world settings. The monetary values derived for each attribute indicated that patients with CKD placed the highest value on the healthcare provider type relative to other attributes. Compared with services provided by a clinical nurse specialist alone, upgrading to services delivered by a combination of an attending nephrologist and a clinical nurse specialist was associated with utility equivalent to an increase in out-of-pocket costs of 52.99 yuan. Compared with services not offering AI-based health services, upgrading to services providing AI-based health features was associated with utility equivalent to an additional out-of-pocket expenditure of 31.44 yuan. Relative to a baseline of one consultation per month, increasing consultation frequency to three times monthly corresponded to utility equivalent to 26.01 yuan in additional out-of-pocket costs, while increasing to five consultations monthly corresponded to 26.15 yuan. Compared with manual entry of health data, upgrading to automated remote monitoring was associated with utility equivalent to 20.48 yuan in additional out-of-pocket expenditure. Relative to services without online community interaction features, upgrading to services providing such interaction was associated with utility equivalent to 14.53 yuan in additional out-of-pocket costs.

**Table 3:** Willingness-to-pay analysis for digital health service attributes among patients with CKD.

Attribute	Willingness to pay (yuan)	*p*	95%*CI*
Healthcare provider			
Clinical nurse specialist → Attending nephrologist	7.10	0.314	−6.73–20.93
Clinical nurse specialist → Attending nephrologist + Clinical nurse specialist	52.99	<0.001	37.84–68.14
Number of consultations			
1 time → 3 times	26.01	<0.001	13.23–38.79
1 time → 5 times	26.15	0.001	10.18–42.12
AI-powered health services			
Poor → Good	31.44	<0.001	22.58–40.30
Monitoring			
Manual entry → Remote monitoring	20.48	0.002	7.21–33.75
Online community interaction			
No → Yes	14.53	0.004	4.75–23.21

### 3.4 Scenario predictions

Using the estimated mixed logit model, we predicted choice probabilities under alternative scenarios. The baseline profile was: clinical nurse specialist, 1 consultation, poor AI-powered health services, manual monitoring, no online community interaction, and 50 yuan monthly out-of-pocket cost. The predicted probability under this baseline was 0.595. Holding all other attributes constant, we varied one attribute at a time. All changes reported below are absolute percentage-point changes. Full results, including predicted probabilities for each scenario, are provided in Supplementary Table S6.

The largest effect was observed for out-of-pocket cost: increasing the monthly cost from 50 to 100 yuan reduced the predicted probability to 0.368 (a decrease of 22.73 percentage points), and increasing it to 200 yuan reduced the probability to 0.229 (a decrease of 36.63 percentage points). Changing the provider from a clinical nurse specialist alone to an attending nephrologist plus a clinical nurse specialist increased the predicted probability by 12.24 percentage points. Other attribute changes produced smaller increases (1.25–7.09 percentage points; see Supplementary Table S6; Figure 2).

**Figure 2:**
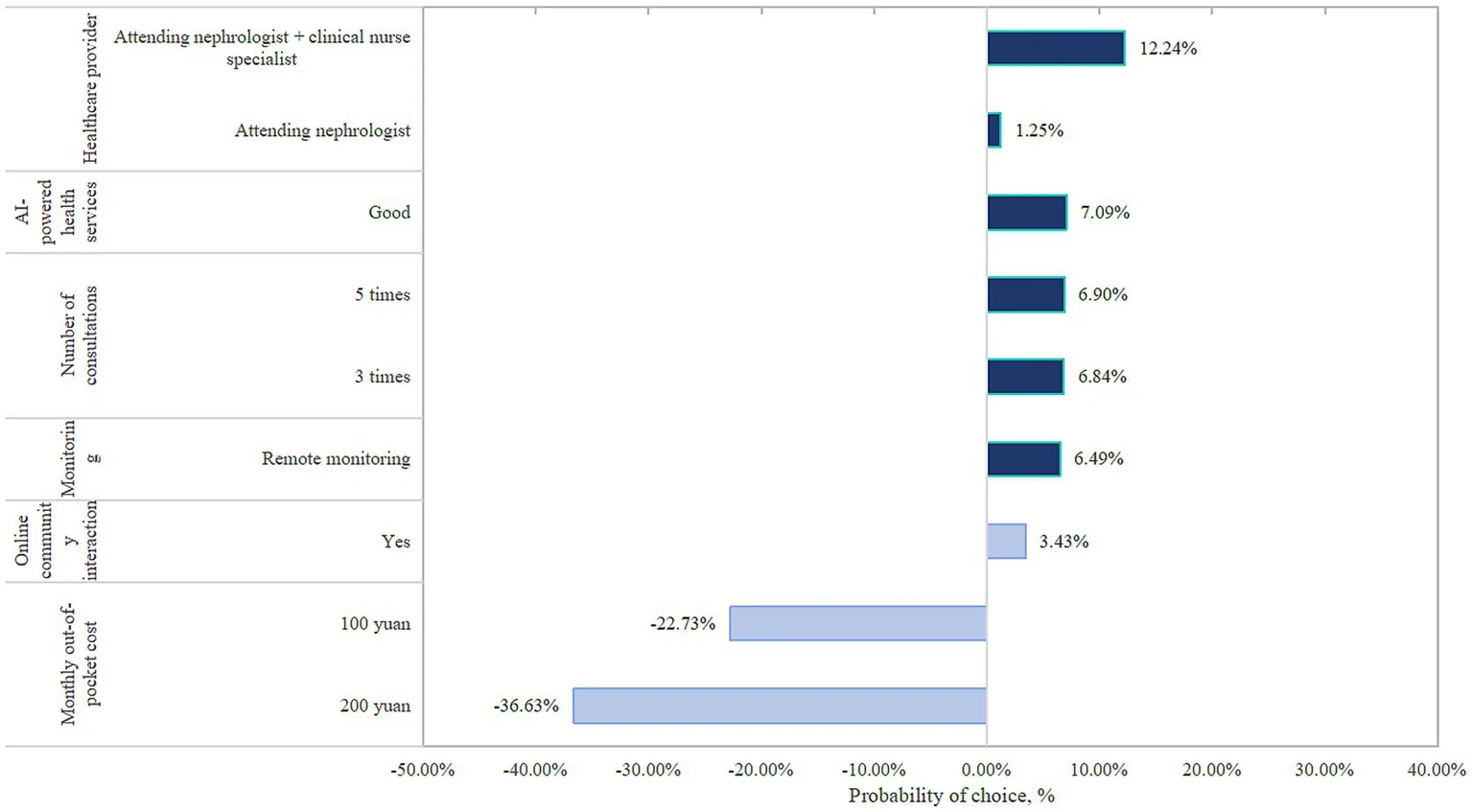
Changes in patients’ choice probabilities under different scenarios.

### 3.5 Subgroup analysis

Exploratory subgroup analyses were conducted using mixed logit regression, with patients with CKD stratified according to individual characteristics, including sex, age, disease duration, and comorbidity categories. Detailed results of subgroup analyses are presented in Supplementary Tables S7–S10.

In these analyses, men showed a stronger preference for an attending nephrologist over a clinical nurse specialist compared with women (difference = 0.452, *p* = 0.015). Among age groups, patients aged <45 years had a stronger aversion to higher out-of-pocket costs (difference = −0.004, *p* = 0.005) and a stronger preference for remote monitoring (difference = 0.553, *p* = 0.006) compared with those aged ≥60 years. Patients aged <45 years also valued AI-powered health services more than those aged 45–60 years (difference = 0.334, *p* = 0.016), but placed less weight on receiving care from an attending nephrologist alone (difference = −0.420, *p* = 0.041). In comorbidity subgroups, patients with hypertension only (Type I) showed a stronger preference for remote monitoring than those with diabetes (Type II; difference = 0.437, *p* = 0.033) or cardiovascular disease (Type III; difference = 0.514, *p* = 0.037). No statistically significant differences were observed for any attribute according to disease duration (all *p* > 0.05). Most other between-group comparisons across sex, age, and comorbidity subgroups were not statistically significant. Willingness-to-pay estimates for digital health service attributes across different subgroups are presented in Figure 3.

**Figure 3:**
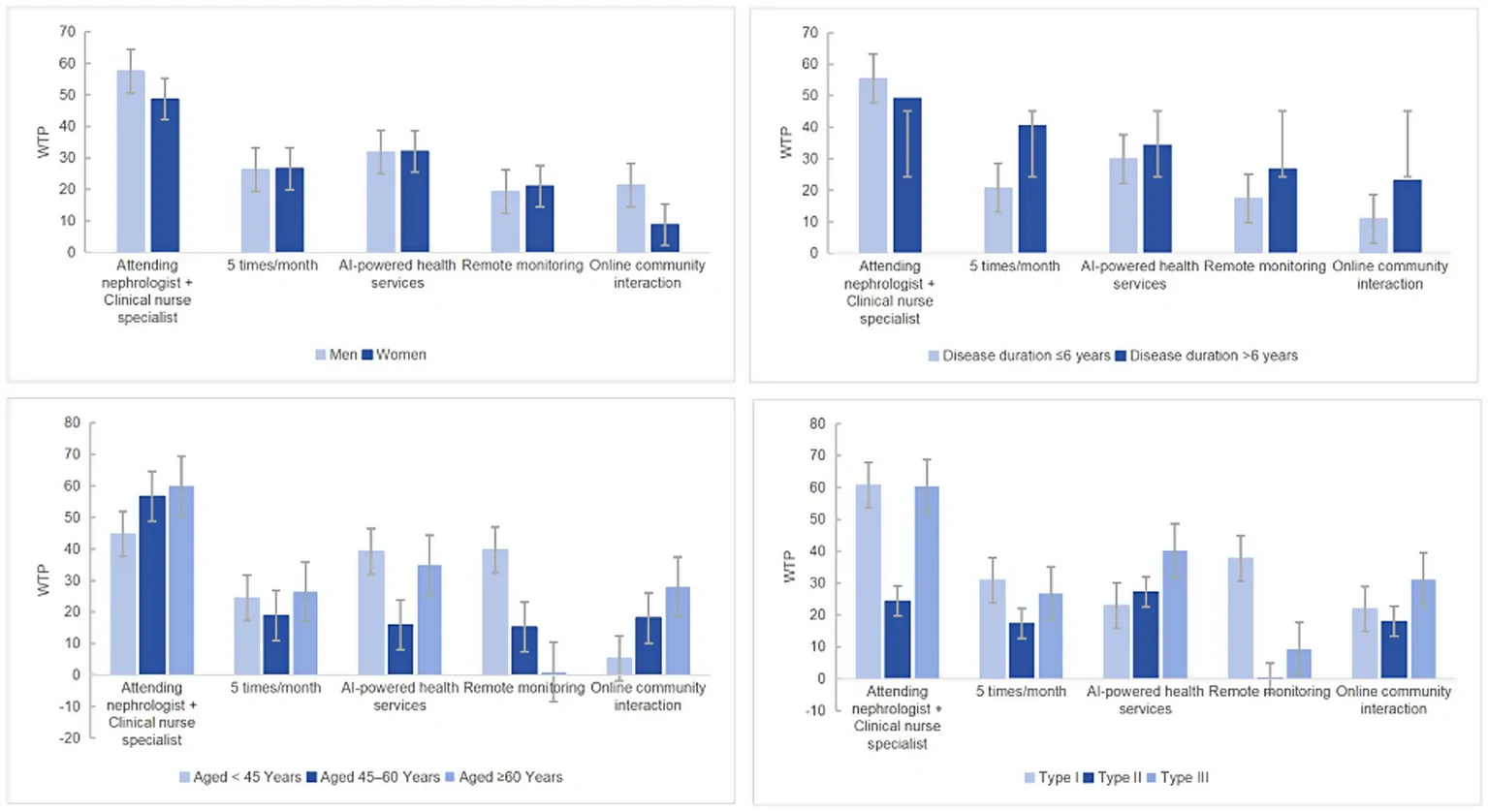
Willingness to pay for each attribute level in subgroup analyses.

## 4 Discussion

Our study employed DCE to quantify patient preferences for digital health services among individuals with chronic kidney disease (CKD). Through a literature review and expert consultations, we identified six attributes that were considered most likely to influence respondents’ preferences: healthcare provider, number of consultations, AI-powered health services, monitoring, online community interaction, and out-of-pocket costs. The results showed that out-of-pocket costs were the most important attribute, followed by healthcare provider and consultation frequency.

This finding is consistent with previous evidence highlighting the substantial economic burden experienced by patients with CKD and the important role of treatment-related expenses in shaping stated healthcare choices in hypothetical choice scenarios (49). Compared with findings from studies conducted in developed countries, the greater relative importance of out-of-pocket costs observed in our study may reflect differences in insurance reimbursement and cost-sharing arrangements, particularly for online health services in China, where patients may bear a greater share of expenses directly. For example, a discrete choice experiment among US patients with CKD found that out-of-pocket costs, although ranked among the three most important attributes, had lower relative importance than that observed in our study (27). This difference may be related to variations in healthcare financing and insurance coverage between the two settings. At the same time, the relative importance assigned to an attribute is influenced by the range of levels over which that attribute is evaluated. Thus, differences in the attributes and level ranges included across DCE may also contribute to variation in relative importance estimates. In our study, the importance assigned to out-of-pocket costs reflects the utility difference associated with the specified range of 50 to 200 yuan and should therefore be interpreted within the context of the experimental design rather than as an absolute measure of the influence of cost on real-world decision-making.

Beyond cost, healthcare provider type was the second most important attribute. Patients preferred a care team comprising attending physicians and specialist nurses over single-provider models. This preference may reflect the complementary roles of physicians and nurses in CKD care, with physicians primarily responsible for diagnosis and treatment, while specialized nurses contribute to follow-up care, patient education, symptom monitoring, and self-management support (50, 51). These findings suggest that digital health services for CKD could incorporate mechanisms that facilitate collaboration and coordination between physicians and nurses, thereby supporting more integrated care delivery (52, 53).

Compared with patients with diabetes, individuals with CKD assigned relatively lower importance to AI-powered health services and monitoring. A discrete choice experiment among patients with diabetes found that AI-powered decision support tools were relatively more important to respondents than AI-powered health services were to participants in our study (54). This difference may reflect variation in preferences for AI-enabled digital health services across chronic disease populations, although differences in DCE design may also have contributed (55). In the context of CKD, the relatively lower importance assigned to AI-powered health services and monitoring may not necessarily indicate a reluctance to use digital technologies, but could instead reflect a preference for digital tools that are integrated with transparent, clinician-linked, and individualized care. Previous studies have suggested that trust in digital health among people with CKD is influenced by familiarity with technology, established relationships with clinicians, system transparency, and the perceived trustworthiness of information sources (15). Similarly, electronic symptom monitoring among patients receiving hemodialysis has been reported to be acceptable when supported by timely clinician feedback, active nursing support, and reliable technology (56). These findings suggest that digital health interventions for CKD may be more acceptable when they complement rather than replace professional judgment and ongoing clinical support. However, because actual technology adoption and use were not directly assessed in our study, these findings should be interpreted as reflecting stated preferences within the DCE context rather than actual adoption behavior.

Notably, online community interaction ranked lowest in relative importance, contrasting with the interest in “virtual care communities” reported in some qualitative studies (57, 58). A qualitative study identified several factors that may influence CKD patients’ willingness to engage with online communities, including privacy concerns, perceived credibility of information, and the adequacy of existing social support networks (59). Several factors may help explain the relatively low importance assigned to this attribute in our study. First, the value of online communities may become more apparent during long-term disease management rather than at the initial stage of adopting digital health services. Second, respondents may primarily perceive digital health services as a means of accessing professional care rather than as a platform for social interaction. Third, the greater reliance on family-based support in the Chinese context may reduce the perceived need for peer support through online communities. These considerations are also consistent with previous research showing that patients may be particularly concerned about the disclosure of identity-related data and the potential commercialization of personal data by private-sector entities, which could further limit their willingness to participate in online communities (60).

Beyond relative importance estimates, scenario predictions indicated that changing the healthcare provider attribute from “clinical nurse specialist” to “attending nephrologist + clinical nurse specialist” was associated with the largest increase in predicted choice probability among non-cost attributes. In contrast, increasing monthly out-of-pocket costs from 50 yuan to 200 yuan was associated with a substantial reduction in predicted choice probability. This suggests strong cost sensitivity but does not imply that actual choice would necessarily decline by this magnitude in real-world settings.

Exploratory subgroup analyses suggested possible differences in preferences across patient groups; however, these analyses were not adjusted for multiple testing and should therefore be viewed as hypothesis-generating rather than confirmatory. Men showed a nominally stronger preference for the type of consultation provider than women, which may reflect gender-specific patterns in health-seeking behaviors and communication styles (61). Younger patients (aged ≤45 years) tended to assign greater relative importance to AI-based health services than older patients, although this pattern was not strictly monotonic; the AI coefficient was highest in the youngest group but overlapped with those of the two older groups. This finding is broadly consistent with previous research indicating greater technology acceptance and higher digital health literacy among younger individuals (62). These exploratory findings could inform future research on stratified or preference-sensitive service design, such as simplified interfaces or additional human support for older patients, but they should not be interpreted as establishing individual-level predictions of preferences.

Patients with a shorter disease duration (≤6 years) showed numerically greater emphasis on the consultation provider and AI-based health services, whereas those with a longer disease duration tended to place greater importance on consultation frequency. However, formal between-group comparisons did not reach statistical significance for any attribute, and these observations should therefore be interpreted with caution. These findings may inform future research on preference-sensitive service design, but they do not currently support stratified service recommendations or individualized care strategies.

Patients in different comorbidity categories showed largely similar preference patterns, with some numerical differences. Formal between-group comparisons did not reach statistical significance for any attribute other than remote monitoring, for which Type I differed significantly from both Type II and Type III. Given the exploratory nature of these analyses and the absence of multiplicity adjustment, this isolated difference should be regarded as hypothesis-generating rather than confirmatory; it may also reflect chance variation or unmeasured clinical heterogeneity. Whether specific comorbidity categories are associated with meaningful differences in preferences remains to be determined in future studies with pre-specified hypotheses and adequate statistical power (63–66). At present, these findings do not support comorbidity-specific service configurations or individualized recommendations.

Several limitations warrant consideration. First, although the sample size was adequate for the main-effects DCE, all participants were recruited from a single center, which may limit the generalizability of the findings to the broader CKD population in China. In addition, the sample size did not permit formal subgroup analyses by CKD stage; future studies with larger, stage-stratified samples are needed to investigate potential stage-specific preferences. Second, the attributes and levels were developed based on a review of the existing literature and expert consultation, without a dedicated qualitative study involving patients. Although a pilot survey was conducted to refine the questionnaire and assess its clarity and comprehensibility, direct patient involvement in attribute development was limited. Moreover, the literature search focused on CKD-specific studies; broader searches across other chronic conditions might have identified additional candidate attributes. As with most DCEs, the six attributes included in this study may not capture all factors relevant to patients’ decision-making. Third, the experimental design did not include an opt-out or status quo alternative, which may have reduced the correspondence between hypothetical choice tasks and real-world decision-making. In addition, confidence intervals for the scenario-based predicted probabilities were not reported because parametric simulation or bootstrap methods were not implemented; incorporating such methods in future studies would help quantify the uncertainty surrounding these predictions. Finally, the exclusion of respondents who failed the dominance or consistency checks or exhibited straight-lining was intended to enhance data quality but may have further reduced the representativeness of the analytic sample. Furthermore, because DCEs elicit stated preferences under hypothetical conditions, the preferences expressed may not fully translate into actual choices or service use in real-world settings.

## 5 Conclusion

The findings of this study indicate that out-of-pocket costs represent the most influential attribute in patients’ stated preferences for digital health services, while the involvement of professional healthcare teams constitutes an important component of service acceptability. These results provide preference evidence that may inform the design of digital health services better aligned with patient priorities. The observed variation in preferences for certain attributes suggests that stratified or preference-sensitive approaches may be worth exploring, although the present data do not establish individual-level preference predictions or confirm the effectiveness of personalized service strategies. Future research should explore whether digital health services designed according to these preference patterns are associated with improved engagement or clinical outcomes in real-world settings.

## Data Availability

The original contributions presented in the study are included in the article/Supplementary material, further inquiries can be directed to the corresponding author.

## References

[1] Maringhini S Zoccali C. Chronic kidney disease progression-a challenge. Biomedicine. (2024) 12:2203. doi: 10.3390/biomedicines12102203, 39457516 PMC11505431

[2] Romagnani P Agarwal R Chan JCN Levin A Kalyesubula R Karam S . Author correction: chronic kidney disease. Nat Rev Dis Primers. (2025) 11:53. doi: 10.1038/s41572-025-00641-2, 40691448

[3] Shabaka S. "From silent damage to inevitable decline: unraveling the progression of CKD". In: Wu HHL, editor. Chronic Kidney Disease - New Updates. Rijeka: IntechOpen (2025).

[4] GBD, Chronic Kidney Disease Collaborators. Global, regional, and national burden of chronic kidney disease in adults, 1990-2023, and its attributable risk factors: a systematic analysis for the global burden of disease study 2023. Lancet. (2023) 406:2461–82. doi: 10.1016/S0140-6736(25)01853-7, 41213283

[5] Wang L Xu X Zhang M Hu C Zhang X Li C . Prevalence of chronic kidney disease in China: results from the sixth China chronic disease and risk factor surveillance. JAMA Intern Med. (2023) 183:298–310. doi: 10.1001/jamainternmed.2022.6817, 36804760 PMC9941971

[6] Chen FY Chang WC Kao ZK Tan AC Li SY Yang CY . Optimizing care for chronic kidney disease: considerations from a to Z. J Chin Med Assoc. (2025) 88:738–46. doi: 10.1097/JCMA.0000000000001280, 40790782 PMC12718738

[7] Safdar F Aslam A. Chronic kidney disease in the primary care setting: a narrative review. J Gen Fam Med. (2025) 26:385–93. doi: 10.1002/jgf2.70054, 40904443 PMC12404170

[8] Combe C Alencar de Pinho N Guedes M Frimat L Fouque D Liabeuf S . Optimal patient care in advanced chronic kidney disease progressing to kidney failure. Nat Rev Nephrol. (2026) 22:406–21. doi: 10.1038/s41581-026-01056-2 41667648

[9] Wallace H Daly JO Jun M Nelson C. Rethinking models of chronic kidney disease care: a narrative review. Kidney Blood Press Res. (2025) 50:433–41. doi: 10.1159/000546562, 40414206 PMC12201929

[10] Deng T Xue Y Methakanjanasak N. Digital health integration in chronic kidney disease. Clin Chim Acta. (2026) 582:120749. doi: 10.1016/j.cca.2025.120749, 41319907

[11] Feng T Li Y Feng Y Zeng C. Application of emerging information technologies in the prevention and control of chronic diseases. Front Public Health. (2026) 14:1755672. doi: 10.3389/fpubh.2026.1755672, 41647744 PMC12868171

[12] Paneerselvam GS Lua PL Chooi WH Rehman IU Goh KW Ming LC. Effectiveness of Mobile apps in improving medication adherence among chronic kidney disease patients: systematic review. J Med Internet Res. (2025) 27:e53144. doi: 10.2196/53144, 40239197 PMC12044313

[13] Bui KL Purtell L Hyun A Bonner A. eHealth solutions for symptom assessment and monitoring in adults with chronic kidney disease: a systematic review. J Clin Nurs. (2025) 34:3560–91. doi: 10.1111/jocn.17827, 40369651 PMC12340751

[14] Zhang K Zhang N Wang R Wei S Ni C. Impact of digital health technology-based nutritional interventions on the nutritional status of hemodialysis patients: a systematic review and meta-analysis. Front Nutr. (2025) 12:1681161. doi: 10.3389/fnut.2025.1681161, 41235312 PMC12604986

[15] Catapan SC Taylor ML Scuffham P Smith AC Kelly JT. Improving consumer trust in digital health: a mixed methods study involving people living with chronic kidney disease. Digit Health. (2025) 11:20552076241312440. doi: 10.1177/20552076241312440, 39801583 PMC11719445

[16] Valkonen P Hölsä S Viitanen J Leinonen S Karisalmi N Rauta V. Illustrating user needs for eHealth with experience map: interview study with chronic kidney disease patients. JMIR Hum Factors. (2025) 12:e48221. doi: 10.2196/48221, 40101207 PMC11962329

[17] Kaklamanou D Nguyen L Al-Abbadey M Sangala N Lewis R. Attitudes towards digital health technology for the care of people with chronic kidney disease: a technology acceptance model exploration. PLOS Digit Health. (2024) 3:e0000614. doi: 10.1371/journal.pdig.0000614, 39383191 PMC11463739

[18] Trần TB Ambrens M Nguyễn J Coleman E Gilanyi Y Letton M . Preferences of people with chronic kidney disease regarding digital health interventions that promote healthy lifestyle: qualitative systematic review with meta-ethnography. BMJ Open. (2024) 14:e082345. doi: 10.1136/bmjopen-2023-082345, 38802278 PMC11131123

[19] Lightfoot CJ Wilkinson TJ Billany RE Sohansoha GK Vadaszy N Ford EC . Use, usability, and experience testing of a digital health intervention to support chronic kidney disease self-management: mixed methods study. J Med Internet Res. (2025) 27:e75845. doi: 10.2196/75845, 40537087 PMC12226781

[20] de Camargo Catapan S Haydon HM Santiago PH Hickman IJ Webb L Isbel N . Development and validation of novel scales to measure trust and confidence in using telephone and video consultation scales in people with chronic kidney disease. J Telemed Telecare. (2026) 32:275–88. doi: 10.1177/1357633X251338950, 40462680

[21] Ryan M Scott DA Reeves C Bate A van Teijlingen ER Russell EM. Eliciting public preferences for healthcare: a systematic review of techniques. Health Technol Assess. (2001) 5:1–186. doi: 10.3310/hta5050, 11262422

[22] Hauber AB González JM Groothuis-Oudshoorn CG Groothuis-Oudshoorn CGM Prior T Marshall DA . Statistical methods for the analysis of discrete choice experiments: a report of the ISPOR conjoint analysis good research practices task force. Value Health. (2016) 19:300–15. doi: 10.1016/j.jval.2016.04.004, 27325321

[23] Clark MD Determann D Petrou S Moro D de Bekker-Grob EW. Discrete choice experiments in health economics: a review of the literature. PharmacoEconomics. (2014) 32:883–902. doi: 10.1007/s40273-014-0170-x, 25005924

[24] Lancsar E Louviere J. Conducting discrete choice experiments to inform healthcare decision making: a user's guide. PharmacoEconomics. (2008) 26:661–77. doi: 10.2165/00019053-200826080-00004, 18620460

[25] Schoot TS van Til J Groothuis-Oudshoorn CKGM. Health outcome preferences and trade-offs among older adults with advanced CKD: a discrete choice experiment. Am J Kidney Dis. (2025) 86:624–633.e1. doi: 10.1053/j.ajkd.2025.06.010 40754291

[26] Wu CH Reed SD Yang JC Mehrotra S McElroy L Gonzalez JM. Do patients think it's worth waiting for a kidney? Evidence from a discrete-choice experiment. Patient. (2026) 19:103–11. doi: 10.1007/s40271-025-00763-5, 40906047 PMC13213703

[27] Walker RC Morton RL Palmer SC Marshall MR Tong A Howard K. A discrete choice study of patient preferences for Dialysis modalities. Clin J Am Soc Nephrol. (2018) 13:100–8. doi: 10.2215/CJN.06830617, 29051145 PMC5753315

[28] Hole B Coast J Caskey FJ Selman LE Rooshenas L Kimpton G . A choice experiment of older patients' preferences for kidney failure treatments. Kidney Int. (2025) 107:130–42. doi: 10.1016/j.kint.2024.08.032, 39368742

[29] Bridges JF Hauber AB Marshall D Lloyd A Prosser LA Regier DA . Conjoint analysis applications in health--a checklist: a report of the ISPOR good research practices for conjoint analysis task force. Value Health. (2011) 14:403–13. doi: 10.1016/j.jval.2010.11.013, 21669364

[30] Kelly JT Jegatheesan DK Dawson J Barnett A Khor BH Chang AR . Are digital health technologies and models of nutrition care the future of chronic kidney disease management? J Ren Nutr. (2023) 33:S80–7. doi: 10.1053/j.jrn.2023.02.004, 36965753

[31] Gc VS Iglesias CP Erdem S Hassan L Peek N Manca A. Using discrete-choice experiments to elicit preferences for digital wearable health technology for self-management of chronic kidney disease. Int J Technol Assess Health Care. (2022) 38:e77. doi: 10.1017/S0266462322003233, 36286261

[32] Yan Y Liu M Duan DF Yan LJ Li L Ma DY. Demand analysis of self-management Mobile health applications for middle-aged and older patients with chronic kidney disease based on the Kano model. Nephron. (2025) 149:1–12. doi: 10.1159/000541729, 39396506

[33] Reed Johnson F Lancsar E Marshall D Kilambi V Mühlbacher A Regier DA . Constructing experimental designs for discrete-choice experiments: report of the ISPOR conjoint analysis experimental design good research practices task force. Value Health. (2013) 16:3–13. doi: 10.1016/j.jval.2012.08.2223, 23337210

[34] Yao L Shang DM Zhao H. The effect and satisfaction of mobile network in the hypertension management of community-dwelling older adults. Chin Gen Pract. (2024) 27:85–90. doi: 10.12114/j.issn.1007-9572.2023.0379

[35] Yang X Ma Y Wang Y Liu M Liu X Jiao X . Effectiveness of mHealth interventions for improving eHealth literacy among patients with chronic diseases: Meta-analysis and systematic review. J Med Internet Res. (2026) 28:e82004. doi: 10.2196/82004, 41996563 PMC13089675

[36] de Bekker-Grob EW Ryan M Gerard K. Discrete choice experiments in health economics: a review of the literature. Health Econ. (2012) 21:145–72. doi: 10.1002/hec.1697, 22223558

[37] Wang H Rowen DL Brazier JE Jiang L. Discrete choice experiments in health state valuation: a systematic review of Progress and new trends. Appl Health Econ Health Policy. (2023) 21:405–18. doi: 10.1007/s40258-023-00794-9, 36997744 PMC10062300

[38] Mulhern B Norman R Street DJ Viney R. One method, many methodological choices: a structured review of discrete-choice experiments for health state valuation. PharmacoEconomics. (2019) 37:29–43. doi: 10.1007/s40273-018-0714-6, 30194624

[39] Hooper R Quintin O Kasza J. Efficient designs for three-sequence stepped wedge trials with continuous recruitment. Clin Trials. (2024) 21:723–33. doi: 10.1177/17407745241251780, 38773924 PMC11528865

[40] Liu Y Kong Q de Bekker-Grob EW. Public preferences for health care facilities in rural China: a discrete choice experiment. Soc Sci Med. (2019) 237:112396. doi: 10.1016/j.socscimed.2019.112396, 31404884

[41] Tervonen T Schmidt-Ott T Marsh K Bridges JFP Quaife M Janssen E. Assessing rationality in discrete choice experiments in health: an investigation into the use of dominance tests. Value Health. (2018) 21:1192–7. doi: 10.1016/j.jval.2018.04.1822, 30314620

[42] Levin A Ahmed SB Carrero JJ Foster B Francis A Hall RK . Executive summary of the KDIGO 2024 clinical practice guideline for the evaluation and Management of Chronic Kidney Disease: known knowns and known unknowns. Kidney Int. (2024) 105:684–701. doi: 10.1016/j.kint.2023.10.016, 38519239

[43] Levey AS Eckardt KU Dorman NM Christiansen SL Hoorn EJ Ingelfinger JR . Nomenclature for kidney function and disease: report of a kidney disease: improving global outcomes (KDIGO) consensus conference. Kidney Int. (2020) 97:1117–29. doi: 10.1016/j.kint.2020.02.010, 32409237

[44] Johnson FR Mansfield C. Survey-design and analytical strategies for better healthcare stated-choice studies. Patient. (2008) 1:299–307. doi: 10.2165/1312067-200801040-00011, 22272997

[45] Yang D Li X Chen B Liu B Huang Z Qin M . Public preferences for preventative dental care: a discrete choice experiment in China. Front Public Health. (2026) 14:1886047. doi: 10.3389/fpubh.2026.1886047, 42741033 PMC13572415

[46] Vass C Boeri M Karim S Marshall D Craig B Ho KA . Accounting for preference heterogeneity in discrete-choice experiments: an ISPOR special interest group report. Value Health. (2022) 25:685–94. doi: 10.1016/j.jval.2022.01.012, 35500943

[47] Ong C Cook AR Tan KK Wang Y. Calculating willingness-to-pay with discrete cost and random coefficients in discrete choice experiments. Heal Econ Rev. (2025) 15:63. doi: 10.1186/s13561-025-00658-z, 40679576 PMC12273327

[48] Quan H Li B Couris CM Fushimi K Graham P Hider P . Updating and validating the Charlson comorbidity index and score for risk adjustment in hospital discharge abstracts using data from 6 countries. Am J Epidemiol. (2011) 173:676–82. doi: 10.1093/aje/kwq433, 21330339

[49] Dodd R Palagyi A Guild L Jha V Jan S. The impact of out-of-pocket costs on treatment commencement and adherence in chronic kidney disease: a systematic review. Health Policy Plan. (2018) 33:1047–54. doi: 10.1093/heapol/czy081, 30247548

[50] Barrett TM Green JA Greer RC Ephraim PL Peskoe S Pendergast JF . Advanced CKD care and decision making: which health care professionals do patients rely on for CKD treatment and advice? Kidney Med. (2020) 2:532–542.e1. doi: 10.1016/j.xkme.2020.05.008, 33089136 PMC7568072

[51] Cahill ML Paulus AB. The impact of interdisciplinary care teams in value-based kidney care: insights from case study reports. Nephrol Nurs J. (2024) 51:413–8. doi: 10.37526/1526-744X.2024.51.5.413, 39503448

[52] Ellis T Kwon AJ Hong MY. The effectiveness of telehealth intervention on chronic kidney disease management in adults: a systematic review. Mayo Clin Proc Digit Health. (2024) 3:100181. doi: 10.1016/j.mcpdig.2024.11.002, 40207003 PMC11975977

[53] Ishani A Christopher J Palmer D Otterness S Clothier B Nugent S . Telehealth by an Interprofessional team in patients with CKD: a randomized controlled trial. Am J Kidney Dis. (2016) 68:41–9. doi: 10.1053/j.ajkd.2016.01.018, 26947216

[54] Ahmad A Khan MU Aslani P. Patient preferences for the treatment of type 2 diabetes in Australia: a discrete choice experiment. J Diabetes Metab Disord. (2022) 21:229–40. doi: 10.1007/s40200-021-00962-5, 35673490 PMC9167383

[55] Shen H van der Kleij R van der Boog PJM Wang W Song X Li Z . Digital tools/eHealth to support CKD self-management: a qualitative study of perceptions, attitudes and needs of patients and health care professionals in China. Int J Med Inform. (2022) 165:104811. doi: 10.1016/j.ijmedinf.2022.104811, 35753175

[56] Viecelli AK Duncanson E Bennett PN D’Antoine M Dansie K Handke W . Perspectives of patients, nurses, and nephrologists about electronic symptom monitoring with feedback in hemodialysis care. Am J Kidney Dis. (2022) 80:215–226.e1. doi: 10.1053/j.ajkd.2021.12.007, 35085687

[57] Du Y Dennis B Ramirez V Li C Wang J Meireles CL. Experiences and disease self-management in individuals living with chronic kidney disease: qualitative analysis of the National Kidney Foundation's online community. BMC Nephrol. (2022) 23:88. doi: 10.1186/s12882-022-02717-7, 35246060 PMC8894554

[58] Coulson N Beach S Buchanan H. Online peer-to-peer health communities for individuals affected by chronic kidney disease: a scoping review protocol. JBI Evid Synth. (2025) 23:372–8. doi: 10.11124/JBIES-23-00500=-523 39344444

[59] Bujanda-Sainz de Murieta A Escalada-Hernández P San Martín-Rodríguez L Soto-Ruiz N. Determinants of the adoption of an online community for people with diabetes: a qualitative study. Sci Rep. (2025) 15:36944. doi: 10.1038/s41598-025-20960-4, 41125712 PMC12546743

[60] Wang G Yi X Fan H. Care preparedness of family caregivers of maintenance hemodialysis patients: a cross-sectional study. Sci Rep. (2024) 14:18369. doi: 10.1038/s41598-024-69502-4, 39112726 PMC11306220

[61] Seidler ZE Benakovic R Wilson MJ McGee MA Fisher K Smith JA . Approaches to engaging men during primary healthcare encounters: a scoping review. Am J Mens Health. (2024) 18:15579883241241090. doi: 10.1177/15579883241241090, 38606788 PMC11010769

[62] Aras S Drakos C Manimangalam V Nasir MA Burns C Smith D . Influencing public acceptance of artificial intelligence (AI) in healthcare delivery. Front Digital Health. (2026) 7:1664345. doi: 10.3389/fdgth.2025.1664345, 41623889 PMC12853031

[63] Priesterroth LS Hermanns N Kulzer B Haak T Ehrmann D. Counting the minutes: perceived diabetes mental load and its associations with technology use and mental disorders. J Diabetes Sci Technol. (2025) 19:830–5. doi: 10.1177/19322968231214271, 37981751 PMC12034985

[64] Shegal M Hu LT Friesen E Minian N Maslej M Rodak T . Conversational agent interventions in diabetes care: a systematic review. Diabetes Res Clin Pract. (2025) 228:112429. doi: 10.1016/j.diabres.2025.112429, 40840786

[65] Mooghali M Stroud AM Yoo DW. Trustworthy and ethical AI-enabled cardiovascular care: a rapid review. BMC Med Inform Decis Mak. (2024) 24:247. doi: 10.1186/s12911-024-02653-6 PMC11373417 39232725

[66] Vo V Woode ME Carter SM Degeling C Chen G. Unravelling public preferences for the use of artificial intelligence Mobile health applications in Australia. Value Health. (2025) 28:1696–704. doi: 10.1016/j.jval.2025.06.013, 40633727

