# Supplementary material for: Preferences for digital health services among patients with chronic kidney disease: a discrete choice experiment
Source: Front Public Health. 2026 Oct 9;14:1905738. doi: 10.3389/fpubh.2026.1905738 (PMC13690723; doi:10.3389/fpubh.2026.1905738)
Supplement: Supplementary file 1 [file Table_1.docx]

**Supplementary Material**

**Content**

[Table S1 Characteristics of attributes and levels included in the main survey.	1](#_Toc16649)

[Table S2 Mixed Logit model analysis of CKD patients preferences.	2](#_Toc8005)

[Table S3 Conditional Logit model of CKD patients preferences.	3](#_Toc25305)

[Table S4 Mixed logit model analysis of CKD patients’ preferences including all respondents (N = 427).	4](#_Toc17525)

[Table S5 Mixed logit model analysis of CKD patients preferences with cost as a continuous variable.	5](#_Toc1290)

[Table S6 Predicted choice probabilities and absolute changes under alternative scenarios.	6](#_Toc5056)

[Table S7 Subgroup analysis of CKD patients preferences according to sex.	7](#_Toc13356)

[Table S8 Subgroup analysis of CKD patients preferences according to disease durations.	8](#_Toc29556)

[Table S9a Subgroup analysis of CKD patients preferences according to age.	9](#_Toc358)

[Table S9b Formal between-group contrasts in preferences by age.	10](#_Toc11234)

[Table S10a Subgroup analysis of CKD patients preferences according to comorbidity categories.	11](#_Toc2370)

[Table S10b Formal between-group contrasts in preferences by comorbidity categories.	13](#_Toc24860)

**Table S1** Characteristics of attributes and levels included in the main survey.

| Attributes | Definition | Levels |
| --- | --- | --- |
| Monthly out-of-pocket expenses | Monthly cost for patients using a kidney disease management app. | 50 yuan |
|  |  | 100 yuan |
|  |  | 200 yuan |
| Healthcare provider | Healthcare professionals who provide health advice to people. | Clinical nurse specialist |
|  |  | Attending nephrologist |
|  |  | Attending nephrologist + Clinical nurse specialist |
| Number of consultations | People could access online consultations with medical staff a certain number of times per month. | 1 time |
|  |  | 3 times |
|  |  | 5 times |
| AI-powered health services | People could access an AI-powered conversation service to consult about their condition and health management. | Good |
|  |  | Poor |
| Monitoring | People monitoring records for tracking health-related data. | Manual entry |
|  |  | Remote monitoring |
| Online community interaction | People could share and communicate with others using this kidney management app. | No |
|  |  | Yes |

**Table S2** Mixed Logit model analysis of CKD patients preferences.

| **Attributes** | ***β*** | ***SE*** | ***P*** | ***95%CI*** | ***SD*** |
| --- | --- | --- | --- | --- | --- |
| Monthly out-of-pocket expenses |  |  |  |  |  |
| 50 yuan | Ref. |  |  |  |  |
| 100 yuan | -1.231 | 0.090 | <0.001 | -1.408 to -1.055 | / |
| 200 yuan | -2.113 | 0.116 | <0.001 | -2.340 to -1.885 | / |
| Monitoring |  |  |  |  |  |
| Manual entry | Ref. |  |  |  |  |
| Remote monitoring | 0.326 | 0.089 | <0.001 | 0.153 to 0.500 | 3.273; *P* <0.001 |
| Healthcare provider |  |  |  |  |  |
| Clinical nurse specialist | Ref. |  |  |  |  |
| Attending nephrologist | 0.078 | 0.093 | 0.400 | -0.104 to 0.261 | 1.719; *P* <0.001 |
| Attending nephrologist + Clinical nurse specialist | 0.716 | 0.105 | <0.001 | 0.510 to 0.921 | 0.979; *P* =0.942 |
| Online community interaction |  |  |  |  |  |
| No | Ref. |  |  |  |  |
| Yes | 0.190 | 0.065 | 0.004 | 0.063 to 0.318 | 0.998; *P* =0.988 |
| AI-powered health services |  |  |  |  |  |
| Poor | Ref. |  |  |  |  |
| Good | 0.401 | 0.063 | <0.001 | 0.277 to 0.524 | 1.004; *P* =0.979 |
| Number of consultations |  |  |  |  |  |
| 1 time | Ref. |  |  |  |  |
| 3 times | 0.386 | 0.086 | <0.001 | 0.217 to 0.555 | 0.954; *P* =0.868 |
| 5 times | 0.414 | 0.105 | <0.001 | 0.208 to 0.620 | 2.689; *P* <0.001 |
| Log likelihood = -1362.1698 |  |  |  |  |  |
| AIC = 2756.340 |  |  |  |  |  |
| BIC = 2861.094 |  |  |  |  |  |

Abbreviations: *β*, the average preferences of the study population; *SE*, standard error; *SD*, standard deviation; Ref, reference level. Standard deviations of random parameters are reported on the original scale. They were obtained by exponentiating the log-standard deviation coefficients from the mixed logit model.

**Table S3** Conditional Logit model of CKD patients preferences.

| **Attributes** | ***β*** | ***SE*** | ***P*** | **95%*CI*** |
| --- | --- | --- | --- | --- |
| Monthly out-of-pocket expenses |  |  |  |  |
| 50 yuan | Ref. |  |  |  |
| 100 yuan | -0.956 | 0.069 | <0.001 | -1.092 to -0.820 |
| 200 yuan | -1.587 | 0.076 | <0.001 | -1.735 to -1.438 |
| Monitoring |  |  |  |  |
| Manual entry | Ref. |  |  |  |
| Remote monitoring | 0.249 | 0.047 | <0.001 | 0.156 to 0.341 |
| Healthcare provider |  |  |  |  |
| Clinical nurse specialist | Ref. |  |  |  |
| Attending nephrologist | 0.053 | 0.066 | 0.420 | -0.076 to 0.183 |
| Attending nephrologist + Clinical nurse specialist | 0.512 | 0.066 | <0.001 | 0.382 to 0.642 |
| Online community interaction |  |  |  |  |
| No | Ref. |  |  |  |
| Yes | 0.147 | 0.048 | 0.002 | 0.053 to 0.241 |
| AI-powered health services |  |  |  |  |
| Poor | Ref. |  |  |  |
| Good | 0.287 | 0.047 | <0.001 | 0.195 to 0.378 |
| Number of consultations |  |  |  |  |
| 1 time | Ref. |  |  |  |
| 3 times | 0.304 | 0.071 | <0.001 | 0.164 to 0.444 |
| 5 times | 0.315 | 0.066 | <0.001 | 0.185 to 0.445 |
| Log likelihood = -1445.3879 |  |  |  |  |
| AIC = 2908.776 |  |  |  |  |
| BIC = 2967.700 |  |  |  |  |

Abbreviations: *β*, the average preferences of the study population; *SE*, standard error; Ref, reference level.

**Table S4** Mixed logit model analysis of CKD patients’ preferences including all respondents (N = 427).

| **Attributes** | ***β*** | ***SE*** | ***P*** | ***95%CI*** | ***SD*** |
| --- | --- | --- | --- | --- | --- |
| Monthly out-of-pocket expenses |  |  |  |  |  |
| 50 yuan | Ref. |  |  |  |  |
| 100 yuan | -1.009 | 0.068 | <0.001 | -1.143 to -0.875 | / |
| 200 yuan | -1.570 | 0.079 | <0.001 | -1.725 to -1.415 | / |
| Monitoring |  |  |  |  |  |
| Manual entry | Ref. |  |  |  |  |
| Remote monitoring | 0.078 | 0.062 | 0.212 | -0.044 to 0.200 | 2.472; *P* <0.001 |
| Healthcare provider |  |  |  |  |  |
| Clinical nurse specialist | Ref. |  |  |  |  |
| Attending nephrologist | -0.069 | 0.069 | 0.313 | -0.204 to 0.065 | 1.419; *P* =0.028 |
| Attending nephrologist + Clinical nurse specialist | 0.461 | 0.076 | <0.001 | 0.312 to 0.610 | 1.000; *P* =0.999 |
| Online community interaction |  |  |  |  |  |
| No | Ref. |  |  |  |  |
| Yes | 0.163 | 0.048 | 0.001 | 0.069 to 0.257 | 1.006; *P* =0.942 |
| AI-powered health services |  |  |  |  |  |
| Poor | Ref. |  |  |  |  |
| Good | 0.435 | 0.048 | <0.001 | 0.341 to 0.530 | 1.183; *P* =0.379 |
| Number of consultations |  |  |  |  |  |
| 1 time | Ref. |  |  |  |  |
| 3 times | 0.409 | 0.067 | <0.001 | 0.279 to 0.540 | 1.003; *P* =0.983 |
| 5 times | 0.371 | 0.073 | <0.001 | 0.229 to 0.513 | 1.665; *P* <0.001 |
| Log likelihood = -1949.7028 |  |  |  |  |  |
| AIC = 3931.406 |  |  |  |  |  |
| BIC = 3996.316 |  |  |  |  |  |

Abbreviations: *β*, the average preferences of the study population; *SE*, standard error; *SD*, standard deviation; Ref, reference level. Standard deviations of random parameters are reported on the original scale. They were obtained by exponentiating the log-standard deviation coefficients from the mixed logit model.

**Table S5** Mixed logit model analysis of CKD patients preferences with cost as a continuous variable.

| **Attributes** | ***β*** | ***SE*** | ***P*** | ***95% CI*** | ***SD*** |
| --- | --- | --- | --- | --- | --- |
| Monthly out-of-pocket expenses | -0.013 | 0.001 | <0.001 | -0.014 to -0.011 | / |
| Monitoring |  |  |  |  |  |
| Manual entry | Ref. |  |  |  |  |
| Remote monitoring | 0.262 | 0.085 | 0.002 | 0.095 to 0.429 | 3.089; *P* <0.001 |
| Healthcare provider |  |  |  |  |  |
| Clinical nurse specialist | Ref. |  |  |  |  |
| Attending nephrologist | 0.091 | 0.091 | 0.317 | -0.087 to 0.269 | 1.594; *P* =0.004 |
| Attending nephrologist + Clinical nurse specialist | 0.678 | 0.102 | <0.001 | 0.479 to 0.877 | 0.990; *P* =0.962 |
| Online community interaction |  |  |  |  |  |
| No | Ref. |  |  |  |  |
| Yes | 0.186 | 0.063 | 0.003 | 0.062 to 0.310 | 0.999; *P* =0.996 |
| AI-powered health services |  |  |  |  |  |
| Poor | Ref. |  |  |  |  |
| Good | 0.402 | 0.061 | <0.001 | 0.282 to 0.523 | 1.005; *P* =0.973 |
| Number of consultations |  |  |  |  |  |
| 1 time | Ref. |  |  |  |  |
| 3 times | 0.333 | 0.083 | <0.001 | 0.171 to 0.495 | 0.972; *P* =0.883 |
| 5 times | 0.334 | 0.105 | 0.001 | 0.129 to 0.539 | 2.815; *P* <0.001 |
| Log likelihood = -1363.2788 |  |  |  |  |  |
| AIC = 2756.558 |  |  |  |  |  |
| BIC = 2854.760 |  |  |  |  |  |

Abbreviations: *β*, the average preferences of the study population; *SE*, standard error; *SD*, standard deviation; Ref, reference level. Standard deviations of random parameters are reported on the original scale. They were obtained by exponentiating the log-standard deviation coefficients from the mixed logit model.

**Table S6** Predicted choice probabilities and absolute changes under alternative scenarios.

| **Attribute** | **Alternative level** | **Predicted probability** | **Absolute change in probability** |
| --- | --- | --- | --- |
| Baseline | — | 0.595 | — |
| Healthcare provider | Attending nephrologist | 0.608 | +1.25 |
| Healthcare provider | Attending nephrologist + clinical nurse specialist | 0.718 | +12.24 |
| Number of consultations | 3 times | 0.663 | +6.84 |
| Number of consultations | 5 times | 0.664 | +6.90 |
| AI-powered health services | Good | 0.666 | +7.09 |
| Monitoring | Remote monitoring | 0.660 | +6.49 |
| Online community interaction | Yes | 0.629 | +3.43 |
| Monthly out-of-pocket cost | 100 yuan | 0.368 | −22.73 |
| Monthly out-of-pocket cost | 200 yuan | 0.229 | −36.63 |

Abbreviations: —, reference level or not applicable.

**Table S7** Subgroup analysis of CKD patients preferences according to sex.

| Attributes | Men (n=144) | |  | Women (n=178) | | Difference | *SE* | 95% *CI* | *P* for sex difference |
| --- | --- | --- | --- | --- | --- | --- | --- | --- | --- |
|  | *β (SE)* | *P* |  | *β (SE)* | *P* |  |  |  |  |
| Monthly out-of-pocket expenses | -0.014(0.001) | <0.001 |  | -0.012(0.001) | <0.001 | -0.002 | 0.001 | -0.005 to 0.001 | 0.157 |
| Monitoring |  |  |  |  |  |  |  |  |  |
| Manual entry | Ref. |  |  | Ref. |  | — | — | — | — |
| Remote monitoring | 0.265(0.123) | 0.032 |  | 0.258(0.120) | 0.031 | 0.007 | 0.172 | -0.330 to 0.344 | 0.968 |
| Healthcare provider |  |  |  |  |  |  |  |  |  |
| Clinical nurse specialist | Ref. |  |  | Ref. |  | — | — | — | — |
| Attending nephrologist | 0.343(0.137) | 0.012 |  | -0.109(0.125) | 0.382 | 0.452 | 0.185 | 0.089 to 0.816 | 0.015 |
| Attending nephrologist + Clinical nurse specialist | 0.786(0.154) | <0.001 |  | 0.599(0.137) | <0.001 | 0.187 | 0.206 | -0.217 to 0.591 | 0.364 |
| Online community interaction |  |  |  |  |  |  |  |  |  |
| No | Ref. |  |  | Ref. |  | — | — | — | — |
| Yes | 0.292(0.096) | 0.002 |  | 0.109(0.086) | 0.204 | 0.183 | 0.129 | -0.070 to 0.436 | 0.156 |
| AI-powered health services |  |  |  |  |  |  |  |  |  |
| Poor | Ref. |  |  | Ref. |  | — | — | — | — |
| Good | 0.437(0.092) | <0.001 |  | 0.394(0.085) | <0.001 | 0.043 | 0.125 | -0.203 to 0.289 | 0.732 |
| Number of consultations |  |  |  |  |  |  |  |  |  |
| 1 time | Ref. |  |  | Ref. |  | — | — | — | — |
| 3 times | 0.410(0.126) | 0.001 |  | 0.269(0.111) | 0.015 | 0.141 | 0.168 | -0.188 to 0.470 | 0.401 |
| 5 times | 0.360(0.159) | 0.023 |  | 0.327(0.142) | 0.021 | 0.033 | 0.213 | -0.385 to 0.451 | 0.877 |

Abbreviations: *β*, the average preferences of the study population; *SE*, standard error; —, reference level or not applicable; Ref, reference level.

**Table S8** Subgroup analysis of CKD patients preferences according to disease durations.

| Attributes | Diseasedurations≤6years(n=208) | |  | Diseasedurations>6years(n=114) | |  | Difference | *SE* | 95%*CI* | *P* for disease durations difference |
| --- | --- | --- | --- | --- | --- | --- | --- | --- | --- | --- |
|  | *β(SE)* | *P* |  | *β(SE)* | *P* |  |  |  |  |  |
| Monthly out-of-pocket expenses | -0.014(0.001) | <0.001 |  | -0.012(0.001) | <0.001 | | -0.002 | 0.0014 | -0.005 to 0.001 | 0.157 |
| Monitoring |  |  |  |  |  | |  |  |  |  |
| Manual entry | Ref. |  |  | Ref. |  | | — | — | — | — |
| Remote monitoring | 0.235(0.103) | 0.023 |  | 0.308(0.153) | 0.044 | | -0.073 | 0.184 | -0.435 to 0.289 | 0.692 |
| Healthcare provider |  |  |  |  |  | |  |  |  |  |
| Clinical nurse specialist | Ref. |  |  | Ref. |  | | — | — | — | — |
| Attending nephrologist | 0.084(0.115) | 0.465 |  | 0.091(0.152) | 0.550 | | -0.007 | 0.191 | -0.381 to 0.367 | 0.971 |
| Attending nephrologist + Clinical nurse specialist | 0.749(0.126) | <0.001 |  | 0.566(0.174) | 0.001 | | 0.183 | 0.215 | -0.238 to 0.604 | 0.395 |
| Online community interaction |  |  |  |  |  | |  |  |  |  |
| No | Ref. |  |  | Ref. |  | | — | — | — | — |
| Yes | 0.148(0.080) | 0.062 |  | 0.267(0.107) | 0.013 | | -0.119 | 0.134 | -0.381 to 0.143 | 0.373 |
| AI-powered health services |  |  |  |  |  | |  |  |  |  |
| Poor | Ref. |  |  | Ref. |  | | — | — | — | — |
| Good | 0.405(0.078) | <0.001 |  | 0.395(0.103) | <0.001 | | 0.010 | 0.129 | -0.243 to 0.263 | 0.938 |
| Number of consultations |  |  |  |  |  | |  |  |  |  |
| 1 time | Ref. |  |  | Ref. |  | | — | — | — | — |
| 3 times | 0.296(0.104) | 0.004 |  | 0.386(0.139) | 0.006 | | -0.090 | 0.174 | -0.430 to 0.250 | 0.604 |
| 5 times | 0.281(0.130) | 0.031 |  | 0.466(0.179) | 0.009 | | -0.185 | 0.221 | -0.619 to 0.249 | 0.403 |

Abbreviations: *β*, the average preferences of the study population; *SE*, standard error; —, reference level or not applicable; Ref, reference level.

**Table S9a** Subgroup analysis of CKD patients preferences according to age.

| Attributes | Age<45 (n=154) | | | | |  | Age 45-60 years (n=94) | | | | |  | Age≥60 years (n=74) | | | | |
| --- | --- | --- | --- | --- | --- | --- | --- | --- | --- | --- | --- | --- | --- | --- | --- | --- | --- |
|  | *β* | *SE* | *P* | 95%*CI* | |  | *β* | *SE* | *P* | 95%*CI* | |  | *β* | *SE* | *P* | 95%*CI* | |
| Monthly out-of-pocket expenses | -0.014 | 0.001 | <0.001 | -0.015 | -0.012 |  | -0.014 | 0.001 | <0.001 | -0.016 | -0.011 |  | -0.010 | 0.001 | <0.001 | -0.012 | -0.008 |
| Monitoring |  |  |  |  |  |  |  |  |  |  |  |  |  |  |  |  |  |
| Manual entry | Ref. |  |  |  |  |  | Ref. |  |  |  |  |  | Ref. |  |  |  |  |
| Remote monitoring | 0.562 | 0.140 | <0.001 | 0.287 | 0.836 |  | 0.211 | 0.140 | 0.131 | -0.063 | 0.484 |  | 0.009 | 0.143 | 0.949 | -0.271 | 0.289 |
| Healthcare provider |  |  |  |  |  |  |  |  |  |  |  |  |  |  |  |  |  |
| Clinical nurse specialist | Ref. |  |  |  |  |  | Ref. |  |  |  |  |  | Ref. |  |  |  |  |
| Attending nephrologist | -0.144 | 0.144 | 0.318 | -0.426 | 0.139 |  | 0.276 | 0.146 | 0.059 | -0.010 | 0.562 |  | 0.267 | 0.163 | 0.102 | -0.053 | 0.586 |
| Attending nephrologist + Clinical nurse specialist | 0.633 | 0.162 | <0.001 | 0.316 | 0.949 |  | 0.784 | 0.163 | <0.001 | 0.465 | 1.103 |  | 0.596 | 0.178 | 0.001 | 0.247 | 0.944 |
| Online community interaction |  |  |  |  |  |  |  |  |  |  |  |  |  |  |  |  |  |
| No | Ref. |  |  |  |  |  | Ref. |  |  |  |  |  | Ref. |  |  |  |  |
| Yes | 0.075 | 0.098 | 0.443 | -0.116 | 0.266 |  | 0.250 | 0.103 | 0.015 | 0.049 | 0.451 |  | 0.278 | 0.117 | 0.018 | 0.048 | 0.507 |
| AI-powered health services |  |  |  |  |  |  |  |  |  |  |  |  |  |  |  |  |  |
| Poor | Ref. |  |  |  |  |  | Ref. |  |  |  |  |  | Ref. |  |  |  |  |
| Good | 0.554 | 0.099 | <0.001 | 0.360 | 0.748 |  | 0.220 | 0.097 | 0.023 | 0.031 | 0.409 |  | 0.346 | 0.111 | 0.002 | 0.129 | 0.563 |
| Number of consultations |  |  |  |  |  |  |  |  |  |  |  |  |  |  |  |  |  |
| 1 time | Ref. |  |  |  |  |  | Ref. |  |  |  |  |  | Ref. |  |  |  |  |
| 3 times | 0.398 | 0.129 | 0.002 | 0.145 | 0.651 |  | 0.177 | 0.132 | 0.182 | -0.083 | 0.436 |  | 0.388 | 0.155 | 0.013 | 0.083 | 0.692 |
| 5 times | 0.346 | 0.167 | 0.038 | 0.019 | 0.673 |  | 0.260 | 0.162 | 0.109 | -0.058 | 0.579 |  | 0.262 | 0.186 | 0.158 | -0.102 | 0.626 |

Abbreviations: *β*, the average preferences of the study population; *SE*, standard error; Ref, reference level.

**Table S9b** Formal between-group contrasts in preferences by age.

| Attributes | Age <45 vs 45–60 | | |  | Age <45 vs ≥60 | | |  | Age 45–60 vs ≥60 | | |
| --- | --- | --- | --- | --- | --- | --- | --- | --- | --- | --- | --- |
|  | Difference (*SE*) | 95%*CI* | *P* |  | Difference (*SE*) | 95%*CI* | *P* |  | Difference (*SE*) | 95%*CI* | *P* |
| Monthly out-of-pocket expenses | 0.000 (0.001) | -0.003 to 0.003 | 1.000 |  | -0.004 (0.001) | -0.007 to -0.001 | 0.005 |  | -0.004 (0.001) | -0.007 to -0.001 | 0.005 |
| Monitoring |  |  |  |  |  |  |  |  |  |  |  |
| Manual entry | — | — | — |  | — | — | — |  | — | — | — |
| Remote monitoring | 0.351 (0.198) | -0.037 to 0.739 | 0.076 |  | 0.553 (0.200) | 0.161 to 0.945 | 0.006 |  | 0.202 (0.200) | -0.190 to 0.594 | 0.313 |
| Healthcare provider |  |  |  |  |  |  |  |  |  |  |  |
| Clinical nurse specialist | — | — | — |  | — | — | — |  | — | — | — |
| Attending nephrologist | -0.420 (0.205) | -0.822 to -0.018 | 0.041 |  | -0.411 (0.218) | -0.837 to 0.015 | 0.059 |  | 0.009 (0.219) | -0.420 to 0.438 | 0.967 |
| Attending nephrologist + Clinical nurse specialist | -0.151 (0.230) | -0.601 to 0.299 | 0.511 |  | 0.037 (0.241) | -0.435 to 0.509 | 0.878 |  | 0.188 (0.241) | -0.285 to 0.661 | 0.436 |
| Online community interaction |  |  |  |  |  |  |  |  |  |  |  |
| No | — | — | — |  | — | — | — |  | — | — | — |
| Yes | -0.175 (0.142) | -0.454 to 0.104 | 0.218 |  | -0.203 (0.153) | -0.502 to 0.096 | 0.184 |  | -0.028 (0.156) | -0.334 to 0.278 | 0.857 |
| AI-powered health services |  |  |  |  |  |  |  |  |  |  |  |
| Poor | — | — | — |  | — | — | — |  | — | — | — |
| Good | 0.334 (0.139) | 0.062 to 0.606 | 0.016 |  | 0.208 (0.149) | -0.083 to 0.499 | 0.162 |  | -0.126 (0.147) | -0.415 to 0.163 | 0.393 |
| Number of consultations |  |  |  |  |  |  |  |  |  |  |  |
| 1 time | — | — | — |  | — | — | — |  | — | — | — |
| 3 times | 0.221 (0.185) | -0.141 to 0.583 | 0.231 |  | 0.010 (0.202) | -0.385 to 0.405 | 0.960 |  | -0.211 (0.204) | -0.610 to 0.188 | 0.300 |
| 5 times | 0.086 (0.233) | -0.370 to 0.542 | 0.711 |  | 0.084 (0.250) | -0.406 to 0.574 | 0.737 |  | -0.002 (0.247) | -0.486 to 0.482 | 0.993 |

Abbreviations: *SE*, standard error; *CI*, confidence interval; —, reference level or not applicable; Ref., reference level.

**Table S10a** Subgroup analysis of CKD patients preferences according to comorbidity categories.

| Attributes | Type Ⅰ (n=168) | | | | |  | Type Ⅱ (n=86) | | | | |  | Type Ⅲ (n=53) | | | | |
| --- | --- | --- | --- | --- | --- | --- | --- | --- | --- | --- | --- | --- | --- | --- | --- | --- | --- |
|  | *β* | *SE* | *P* | 95%*CI* | |  | *β* | *SE* | *P* | 95%*CI* | |  | *β* | *SE* | *P* | 95%*CI* | |
| Monthly out-of-pocket expenses | -0.012 | 0.001 | <0.001 | -0.013 | -0.010 |  | -0.012 | 0.001 | <0.001 | -0.014 | -0.009 |  | -0.008 | 0.002 | <0.001 | -0.011 | -0.005 |
| Monitoring |  |  |  |  |  |  |  |  |  |  |  |  |  |  |  |  |  |
| Manual entry | Ref. |  |  |  |  |  | Ref. |  |  |  |  |  | Ref. |  |  |  |  |
| Remote monitoring | 0.439 | 0.123 | <0.001 | 0.199 | 0.680 |  | 0.002 | 0.164 | 0.989 | -0.319 | 0.324 |  | -0.075 | 0.213 | 0.726 | -0.492 | 0.342 |
| Healthcare provider |  |  |  |  |  |  |  |  |  |  |  |  |  |  |  |  |  |
| Clinical nurse specialist | Ref. |  |  |  |  |  | Ref. |  |  |  |  |  | Ref. |  |  |  |  |
| Attending nephrologist | 0.160 | 0.119 | 0.179 | -0.073 | 0.392 |  | -0.144 | 0.177 | 0.416 | -0.491 | 0.203 |  | 0.217 | 0.227 | 0.340 | -0.229 | 0.663 |
| Attending nephrologist + Clinical nurse specialist | 0.707 | 0.139 | <0.001 | 0.434 | 0.980 |  | 0.286 | 0.196 | 0.143 | -0.097 | 0.670 |  | 0.481 | 0.253 | 0.057 | -0.014 | 0.977 |
| Online community interaction |  |  |  |  |  |  |  |  |  |  |  |  |  |  |  |  |  |
| No | Ref. |  |  |  |  |  | Ref. |  |  |  |  |  | Ref. |  |  |  |  |
| Yes | 0.255 | 0.086 | 0.003 | 0.085 | 0.424 |  | 0.211 | 0.131 | 0.107 | -0.046 | 0.468 |  | 0.249 | 0.163 | 0.126 | -0.070 | 0.567 |
| AI-powered health services |  |  |  |  |  |  |  |  |  |  |  |  |  |  |  |  |  |
| Poor | Ref. |  |  |  |  |  | Ref. |  |  |  |  |  | Ref. |  |  |  |  |
| Good | 0.267 | 0.081 | 0.001 | 0.108 | 0.426 |  | 0.320 | 0.123 | 0.010 | 0.078 | 0.562 |  | 0.321 | 0.156 | 0.040 | 0.015 | 0.627 |
| Number of consultations |  |  |  |  |  |  |  |  |  |  |  |  |  |  |  |  |  |
| 1 time | Ref. |  |  |  |  |  | Ref. |  |  |  |  |  | Ref. |  |  |  |  |
| 3 times | 0.292 | 0.112 | 0.009 | 0.072 | 0.511 |  | 0.245 | 0.168 | 0.144 | -0.084 | 0.575 |  | 0.213 | 0.209 | 0.308 | -0.197 | 0.623 |
| 5 times | 0.360 | 0.140 | 0.010 | 0.086 | 0.633 |  | 0.204 | 0.211 | 0.336 | -0.211 | 0.618 |  | 0.163 | 0.244 | 0.507 | -0.315 | 0.640 |

Abbreviations: *β*, the average preferences of the study population; *CI*, confidence interval; *SE*, standard error; Ref, reference level; Type I, CKD complicated with hypertension; Type II, CKD combined with type 2 diabetes; Type III, CKD combined with cardiovascular diseases, comorbidity groups were mutually exclusive; patients with multiple comorbidities were assigned to a single group based on a prespecified hierarchy (cardiovascular disease > diabetes > hypertension). Among the 322 respondents in the main analysis, 307 had at least one of the three prespecified comorbidities and were included in this subgroup analysis. The remaining 15 respondents had none of these comorbidities and were therefore not assigned to any comorbidity category.

**Table S10b** Formal between-group contrasts in preferences by comorbidity categories.

| Attributes | Type Ⅰ vs Type Ⅱ | | |  | Type Ⅰ vs Type Ⅲ | | |  | Type Ⅱ vs Type Ⅲ | | |
| --- | --- | --- | --- | --- | --- | --- | --- | --- | --- | --- | --- |
|  | Difference (*SE*) | 95%*CI* | *P* |  | Difference (*SE*) | 95%*CI* | *P* |  | Difference (*SE*) | 95%*CI* | *P* |
| Monthly out-of-pocket expenses | 0.000 (0.001) | -0.003 to 0.003 | 1.000 |  | -0.004 (0.002) | -0.008 to 0.000 | 0.074 |  | -0.004 (0.002) | -0.008 to 0.000 | 0.074 |
| Monitoring |  |  |  |  |  |  |  |  |  |  |  |
| Manual entry | — | — | — |  | — | — | — |  | — | — | — |
| Remote monitoring | 0.437 (0.205) | 0.035 to 0.839 | 0.033 |  | 0.514 (0.246) | 0.032 to 0.996 | 0.037 |  | 0.077 (0.269) | -0.450 to 0.604 | 0.775 |
| Healthcare provider |  |  |  |  |  |  |  |  |  |  |  |
| Clinical nurse specialist | — | — | — |  | — | — | — |  | — | — | — |
| Attending nephrologist | 0.304 (0.213) | -0.114 to 0.722 | 0.154 |  | -0.057 (0.256) | -0.559 to 0.445 | 0.824 |  | -0.361 (0.288) | -0.925 to 0.203 | 0.210 |
| Attending nephrologist + Clinical nurse specialist | 0.421 (0.240) | -0.050 to 0.892 | 0.080 |  | 0.226 (0.289) | -0.340 to 0.792 | 0.434 |  | -0.195 (0.320) | -0.822 to 0.432 | 0.542 |
| Online community interaction |  |  |  |  |  |  |  |  |  |  |  |
| No | — | — | — |  | — | — | — |  | — | — | — |
| Yes | 0.044 (0.157) | -0.263 to 0.351 | 0.779 |  | 0.006 (0.184) | -0.355 to 0.367 | 0.974 |  | -0.038 (0.209) | -0.448 to 0.372 | 0.856 |
| AI-powered health services |  |  |  |  |  |  |  |  |  |  |  |
| Poor | — | — | — |  | — | — | — |  | — | — | — |
| Good | -0.053 (0.147) | -0.342 to 0.236 | 0.719 |  | -0.054 (0.176) | -0.399 to 0.291 | 0.759 |  | -0.001 (0.199) | -0.390 to 0.388 | 0.996 |
| Number of consultations |  |  |  |  |  |  |  |  |  |  |  |
| 1 time | — | — | — |  | — | — | — |  | — | — | — |
| 3 times | 0.047 (0.202) | -0.349 to 0.443 | 0.816 |  | 0.079 (0.237) | -0.386 to 0.544 | 0.739 |  | 0.032 (0.268) | -0.494 to 0.558 | 0.905 |
| 5 times | 0.156 (0.253) | -0.340 to 0.652 | 0.538 |  | 0.197 (0.281) | -0.354 to 0.748 | 0.484 |  | 0.041 (0.323) | -0.591 to 0.673 | 0.899 |

Abbreviations: *SE*, standard error; *CI*, confidence interval; —, reference level or not applicable; Type I, CKD complicated with hypertension; Type II, CKD combined with type 2 diabetes; Type III, CKD combined with cardiovascular diseases. Among the 322 respondents in the main analysis, 307 had at least one of the three prespecified comorbidities and were included in the comorbidity subgroup analysis. The remaining 15 respondents had none of these comorbidities and were therefore not assigned to any comorbidity category.
